# Clinical case-study describing the use of skin-perichondrium-cartilage graft from the auricular concha to cover large defects of the nose

**DOI:** 10.1186/1746-160X-8-10

**Published:** 2012-03-19

**Authors:** Francesco Inchingolo, Marco Tatullo, Massimo Marrelli, Alessio D Inchingolo, Roberto Corelli, Angelo M Inchingolo, Gianna Dipalma, Fabio M Abenavoli

**Affiliations:** 1 Department of Dental Sciences and Surgery, University of Bari, Piazza Giulio Cesare, Policlinico, 70124 Bari, Italy; 2 Department of Basic Medical Sciences, University of Bari, Bari, Italy; 3 Department of "Head and Neck Diseases", Hospital "Fatebenefratelli", Rome, Italy; 4 Unit of Maxillofacial Surgery, Calabrodental clinic, Crotone, Italy; 5 Dental School, University of Bari, Bari, Italy; 6 Department of Maxillofacial Surgery, General Hospital, Bari, Italy; 7 Department of Surgical, Reconstructive and Diagnostic Sciences, General Hospital, Milan, Italy; 8 Tecnologica Research Institute, Medical section, Crotone, Italy

## Abstract

**Background:**

The composite graft from the conchal cartilage is a graft that is often used, especially in surgery on the nose, due to its capacity to resolve problems of cover and tissue deficit, arising from the removal of neoplasms or as the result of trauma, burns or following over-aggressive rhinoplasty. We have started to use skin-perichondrium-cartilage graft from the ear to cover large areas of the nose with very satisfying results as well as we describe in the reported clinical case.

**Methods:**

The operation consisted of reconstruction of the cartilaginous nasal septum, which had previously been removed, using two vestibular labial mucosa flaps to reconstruct the mucosa, and cartilage from the ear conch for the cartilaginous septum. After this, the skin edges of the fistula were turned to recreate the inner lining of the nose and form a vascular base of wide area to accept the composite graft. The case concerns a female 74-year old patient who had undergone several oncological surgery for a relapsing basal cell carcinoma on the dorsum of the nose. The operation consisted of reconstruction of the cartilaginous nasal septum using two vestibular labial mucosa flaps to reconstruct the mucosa, and cartilage from the ear conch for the cartilaginous septum.

**Results:**

The perichondrial cutaneous graft has shown in this surgical case very favorable peculiarities that make it usable even in facial plastic surgery.

**Conclusions:**

We believe that the positive experience that we achieved in the use of composite grafts for the reconstruction of large areas of the nose could be interesting for others surgeons.

## Introduction

The composite graft from the conchal cartilage is a graft that is often used, especially in surgery on the nose, due to its capacity to resolve problems of cover and tissue deficit, arising from the removal of neoplasms or as the result of trauma, burns or following over-aggressive rhinoplasty [1-3].

Complex defects of the nose are aesthetically difficult to repair, however, the colour, the quality and thickness of the composite-skin graft harvested from the preauricular site compare favourably with the skin of the nose region, even after extensive oncological surgery such as in patients affected by basal cell carcinoma of the nose [4].

The technique for removal of the graft is very simple, leaves no trace and does not cause any residual functional deficit [5,6]. Insertion of the graft is easy and allows rapid solution of surgical problems that would otherwise demand more lengthy reconstruction or the use of microsurgery.

Working from this point of view, in the last 5 years, we have started to use skin-perichondrium-cartilage graft from the ear to cover large areas of the nose with very satisfying results.

We would like to describe a clinical case-study which we believe can be useful to illustrate our technique.

## Case report

**Figure 1:**
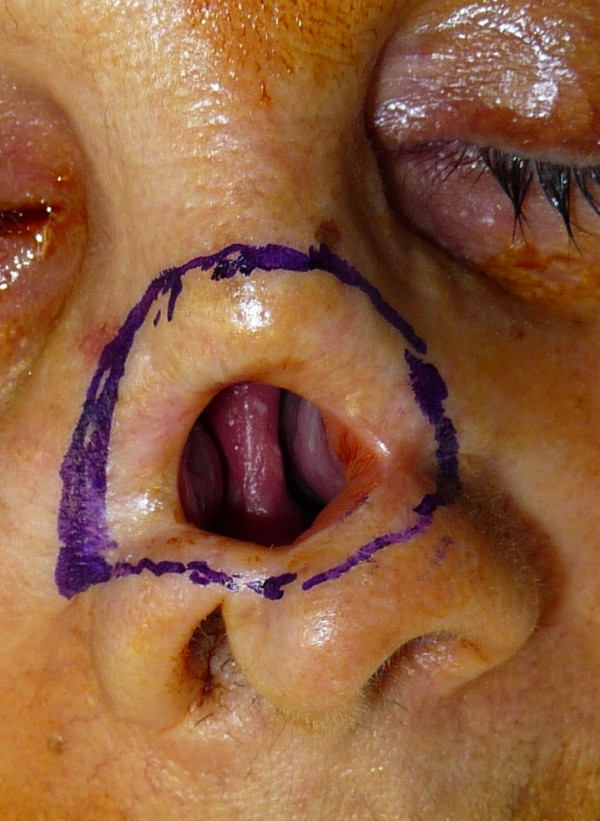
**A preoperative image of the large defect on the dorsum of the nose**.

The case concerns a female 74-year old patient who had undergone several oncological surgery for a relapsing basal cell carcinoma on the dorsum of the nose. It was three years after the last operation and the patient was very keen on a definitive covering of the affected area (Figure 1), which had been made with an epiphysis but which she was no longer able to tolerate. Various reconstructive surgical operations had been proposed to the patient, but she had always refused them.

**Figure 2:**
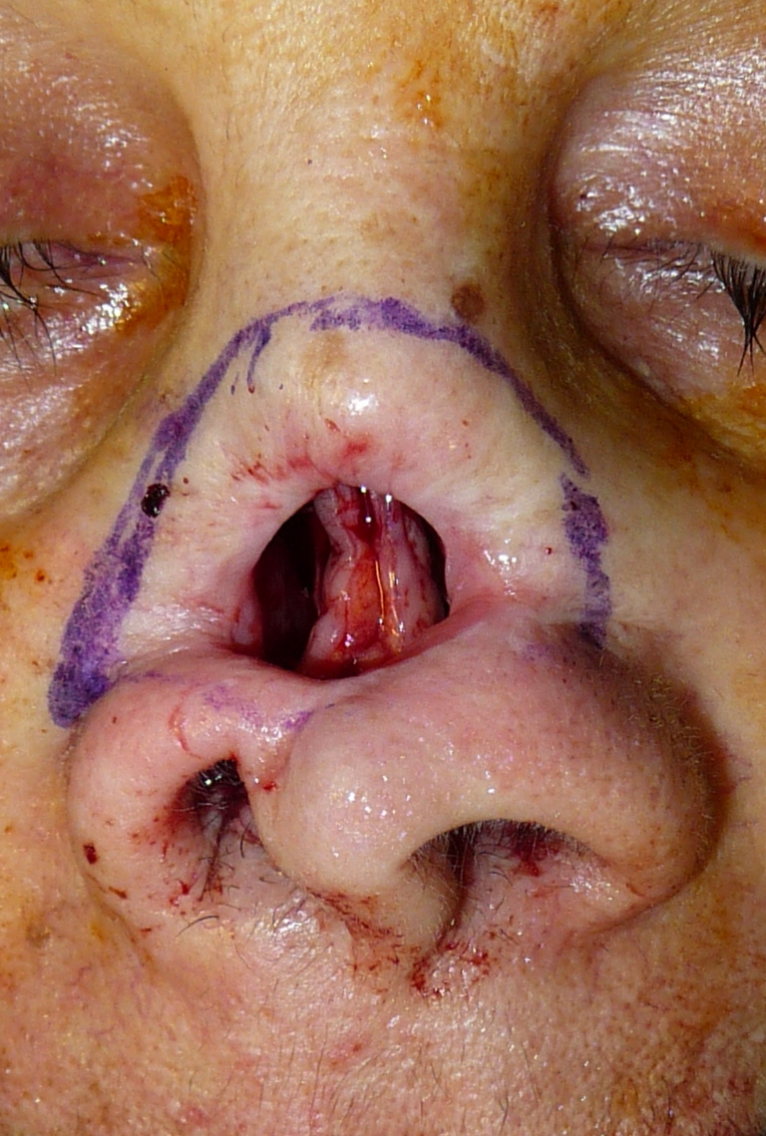
**The cartilaginous nasal septum reconstructed using two vestibular labial mucosa flaps to recreate the mucosa**.

**Figure 3:**
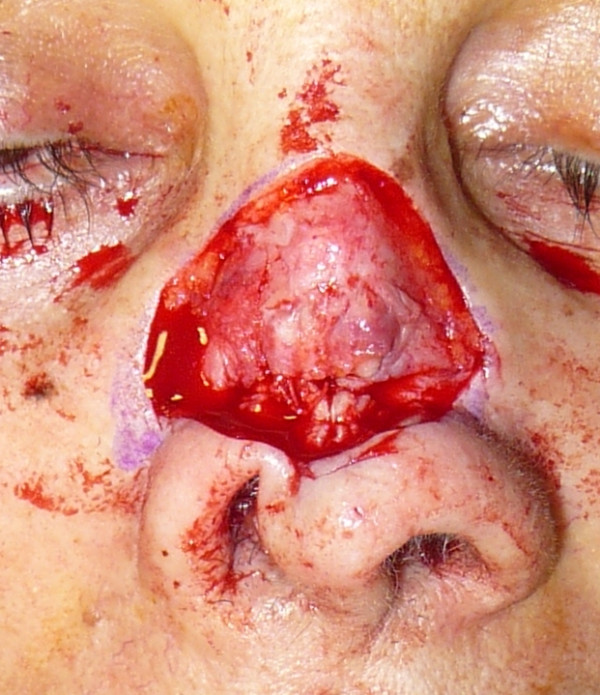
**A view of the skin edges of the fistula turned to recreate the inner lining of the nose**.

**Figure 4:**
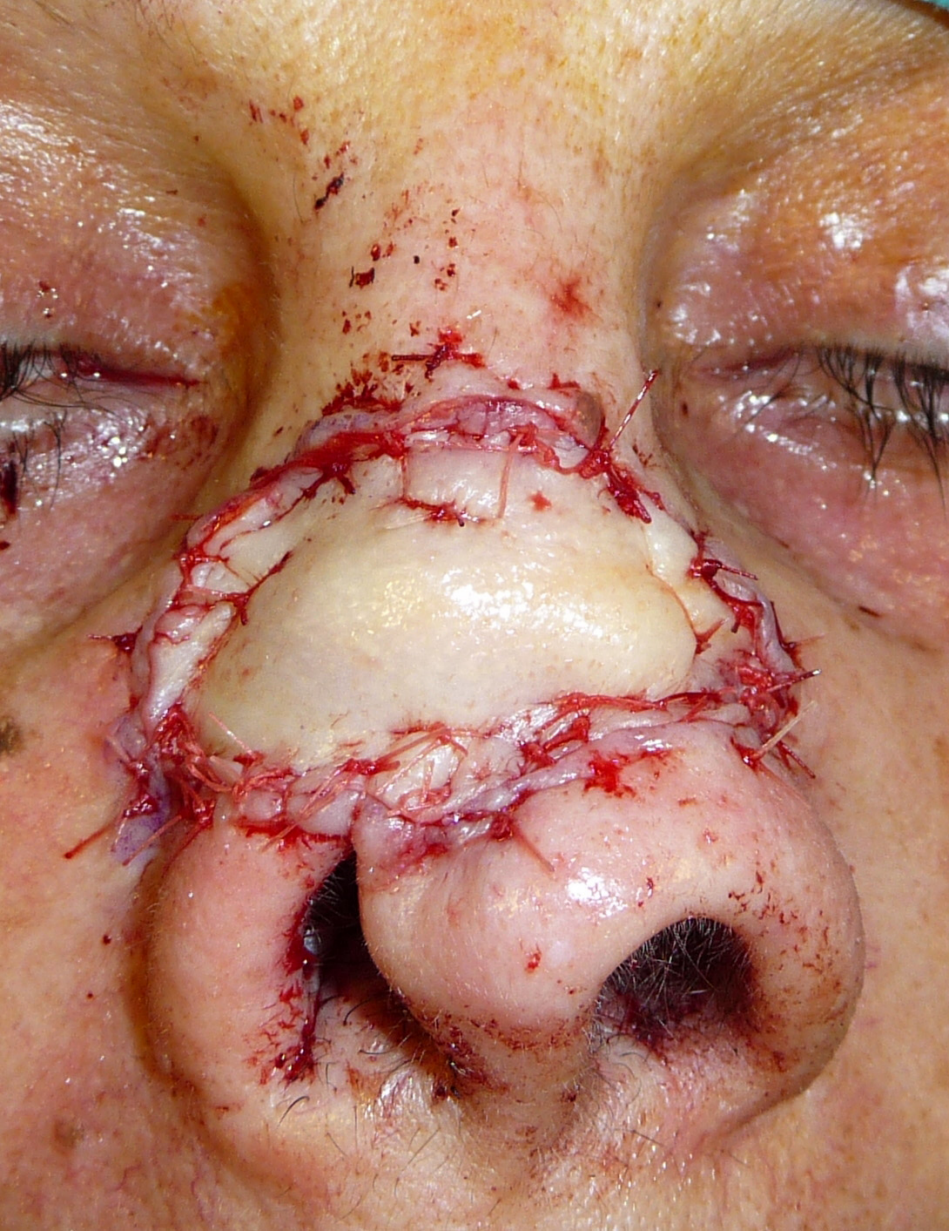
**The surgical area covered by a graft of skin-perichondrium-cartilage removed from the auricular concha**.

**Figure 5:**
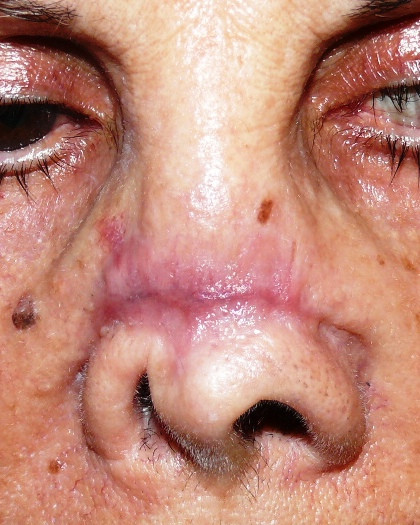
**A postoperative image after about 4 weeks from surgery**.

The operation consisted of reconstruction of the cartilaginous nasal septum, which had previously been removed, using two vestibular labial mucosa flaps to reconstruct the mucosa, and cartilage from the ear conch for the cartilaginous septum (Figure 2). After this, the skin edges of the fistula were turned to recreate the inner lining of the nose and form a vascular base of wide area to accept the composite graft (Figure 3,4). The post-operatory period was regular apart from the central section of the skin graft, which presented a severe inflammation and which was treated with local application of a ointment and with an administration of bromeline pills in order to reduce the swelling [7], giving complete healing within a few weeks (Figure 5).

## Discussion

The perichondrial cutaneous graft is a performant and versatile composite graft that shows useful properties for maxillofacial surgery. The graft consists of epidermis, dermis and subcutaneous tissue, and the perichondrial layer.

The perichondrial cutaneous graft has very favorable peculiarities that make it usable in different types of plastic surgery, in some cases we can certainly use it even in facial plastic surgery. The auricular concha is easily accessible, has a good tissue consistency and adaptability. Useful characteristics are that it does not contract and we can see a low donor site morbidity [8]. However, the color match is not really good in colored races, so we can't suggest this graft in all patients.

One limitation of the technique is determined by the area that can be considered to be sure of proper establishment and revascularisation of the composite graft.

This constraint is however more a subjective limitation than an objective one, since there are no rules that can indicate it to us. What is considered to be important is that the graft should have the maximum possible contact with the vascularised tissue, which can be considered to be the bed upon which the graft is placed; postoperative gentle scarification of the graft, in combination with a constantly applied heparin solution decongests venous stasis normally seen in such grafts helps to establish a stable and early blood supply enhancing graft take [1,9]. Important is then to have the consciousness that creating a well-vascularized recipient bed with an optimization of the raw contact surface give the chance of good result.

## Conclusions

We believe that the positive experience that we achieved in the use of composite grafts for the reconstruction of large areas of the nose could be interesting for others surgeons and will stimulate wider application of the technique described in this report.

## Consent statement

Written informed consent was obtained from the patient for publication of this case report and accompanying images. A copy of the written consent is available for review by the Editor-in-Chief of this journal.

## Competing interests

The authors declare that they have no competing interests.

## Authors' contributions

FI: participated in the surgical treatment and in the follow-up of this patient, MT: drafted the manuscript and reviewed the literature, FMA: participated in the surgical treatment and in the follow-up of this patient, MM: participated in the design of this case study and in the follow-up of this patient, ADI: revised the literature sources, RC: participated in the surgical treatment and in the follow-up of this patient, AMI: documented this case report with digital pictures, GD: participated in the follow-up of this patient. All the authors read and approved the final manuscript.
